# The Use of Expressive Writing in Healthcare Professionals: A Systematic Review of Quantitative Studies

**DOI:** 10.3390/healthcare14081057

**Published:** 2026-04-16

**Authors:** Massimo Guasconi, Federico Dibennardo, Chiara Cosentino, Giovanna Artioli, Angela Andriollo, Sara Pressi, Michela Rocchi, Sarah Santona Galli, Giulia Valente, Antonio Bonacaro

**Affiliations:** 1 Department of Medicine and Surgery, University of Parma, 43125 Parma, Italy; massimo.guasconi@unipr.it (M.G.); giovanna.artioli@unipr.it (G.A.); 2 Azienda USL of Piacenza, 29121 Piacenza, Italy; 3 Independent Consultant, 43126 Parma, Italy; fede.psicologo@gmail.com; 4 Private Practitioner, 1000 Bruxelles, Belgium; chiara.cosentino87@gmail.com; 5 Azienda PSS of Trento, 38123 Trento, Italy; angela.andriollo@apss.tn.it; 6 ASU Giuliano Isontina, 34128 Trieste, Italy; sara.pressi@asugi.sanita.fvg.it; 7 ASST “Sette Laghi”, 21100 Varese, Italy; michela.rocchi@asst-settelaghi.it; 8 “Associazione Triangolo”, 6900 Lugano, Switzerland; sarah.galli@triangolo.ch; 9 ASST “Santi Paolo e Carlo”, 20146 Milano, Italy; giuls.valente@gmail.com

**Keywords:** health personnel, healthcare worker, nurses, doctor, expressive writing, emotional disclosure

## Abstract

Background: Healthcare professionals are exposed to high emotional demands, including repeated contact with suffering, death, moral distress, and organizational pressure. These factors are associated with psychological distress, burnout, and secondary traumatic stress. Expressive Writing (EW) has been proposed as a psychological intervention, but evidence of its effectiveness among healthcare professionals remains heterogeneous. Objectives: To examine the effects of EW on psychological health, psychophysical well-being, and professional satisfaction among healthcare professionals. Methods: A systematic review was conducted in accordance with the Cochrane Handbook for Systematic Reviews of Interventions and the PRISMA 2020 guidelines. Searches were performed in PubMed, CINAHL, CENRAL, CENTRAL Scopus, Embase, and PsycINFO from database inception to January 2025. Quantitative studies involving healthcare professionals and evaluating structured expressive writing interventions were considered for inclusion, including randomized and non-randomized, controlled and uncontrolled designs. Studies reporting psychological, psychophysical, or work-related outcomes were eligible. Only full-text articles published in English or Italian were considered. The review protocol was registered and archived in the Open Science Framework. Methodological quality was assessed using CASP checklists, the RoB 2 tool, and the Newcastle–Ottawa Scale. Results: Seven studies published between 2017 and 2023 were included. EW interventions were associated with reductions in psychological distress, particularly perceived stress, depressive symptoms, and post-traumatic stress symptoms. Findings regarding burnout and compassion fatigue were mixed. Organizational and job-related outcomes, such as job satisfaction and organizational commitment, showed limited and heterogeneous improvements. No consistent effects were observed for resilience or social support. Overall, the methodological quality of the included studies was generally good. Conclusions: EW appears to be a promising, low-cost intervention for reducing psychological distress among healthcare professionals. However, heterogeneity in study designs, intervention protocols, and outcome measures limits the strength of the evidence. Further high-quality, controlled studies using standardized EW protocols are needed.

## 1. Introduction

Healthcare professionals are frequently exposed to stressful and emotionally demanding situations, including the communication of negative diagnoses, repeated contact with suffering and death, exposure to physical or psychological aggression, and sustained high workloads. This burden may be particularly pronounced in critical care and palliative care settings [1]. Continuous exposure to suffering and death may contribute to burnout, compassion fatigue, and secondary traumatic stress [2]. Work-related stress can negatively affect both the health of professionals and the organizations in which they work, with relevant clinical, organizational, and economic implications. For this reason, identifying accessible and potentially effective interventions to reduce psychological distress and promote emotional awareness and reflection is of particular relevance in healthcare settings [3]. Although several psychological interventions may help reduce distress and improve well-being, their implementation in healthcare settings may be limited by time constraints, organizational burden, and the difficulty some professionals experience in openly disclosing emotionally charged experiences in interpersonal contexts [4]. For this reason, writing-based interventions have increasingly been considered potentially feasible and low-threshold approaches for emotional processing and self-reflection [4,5]. Expressive Writing (EW), originally developed within the Pennebaker paradigm, is a structured writing intervention in which participants are typically invited to write about emotionally significant or traumatic experiences for 15–30 min across several consecutive days, usually over 3–5 sessions [4]. Participants are encouraged to focus on their deepest thoughts and emotions related to stressful or traumatic events, with the aim of facilitating emotional disclosure, cognitive processing, and meaning-making [4]. Theoretical accounts suggest that these effects may be mediated by reduced emotional inhibition, repeated exposure to emotionally salient material, and the narrative organization of stressful experiences, which may facilitate self-regulation and cognitive integration [4,5]. Previous research has suggested that engaging in this type of structured emotional writing may be associated with improvements in both psychological and physical health outcomes, as assessed through subjective and objective indicators [4].

Although EW has its origins in clinical psychology, subsequent studies have suggested that it may also be applied in professional contexts, for example, by reducing work-related stress and depressive symptoms and by supporting personal and work-related resources, such as resilience, self-determination, organizational skills, and work commitment [5]. A recent literature review by Lukenda et al. [5] highlighted the potential of expressive writing as a workplace intervention for work-related stress, while Cowen et al. [6] emphasized the broader pedagogical and reflective value of creative and expressive writing in medical education. Together, these findings suggest that writing-based approaches may support both emotional regulation and professional development in healthcare-related contexts [5,6]. However, existing reviews have generally addressed broader writing-based interventions, workplace stress, or educational contexts, rather than providing a focused synthesis of structured expressive writing protocols and their quantitative effects specifically among healthcare professionals. Writing may also foster reflective and expressive skills that are relevant to healthcare practice, including the ability to articulate personal experiences, observe and describe emotionally salient events, and support the development of relational and transversal competencies involved in clinical thinking and professional functioning [6]. Although numerous studies have explored the use of expressive writing in healthcare, the available evidence remains fragmented, often based on small samples and characterized by substantial methodological heterogeneity. In particular, findings are dispersed across different healthcare settings and outcome domains, including psychological distress, burnout-related symptoms, and organizational variables, making it difficult to draw clear conclusions about the role of structured expressive writing interventions in healthcare professionals [1,7,8,9,10,11,12]. In this context, a systematic synthesis of the available quantitative evidence is needed to summarize and integrate current findings on the effects of structured EW protocols in healthcare professionals.

## 2. Materials and Methods

The present systematic review of the literature was conducted to examine the effects on psychological health, psychophysical well-being, and professional satisfaction among healthcare professionals and healthcare students. The study design was informed by the Cochrane Handbook for Systematic Reviews of Interventions [13] and by the PICO framework, adapted to the characteristics of the available literature. The reference population included healthcare professionals and healthcare students; the intervention of interest was EW; no specific comparator was required for eligibility, as both controlled and uncontrolled quantitative study designs were considered; and the outcomes included indicators of psychological health, psychophysical well-being, and perceived work-related functioning. The review protocol was developed in accordance with the Preferred Reporting Items for Systematic Reviews and Meta-Analyses for Protocols (PRISMA-P) [14], in order to enhance methodological transparency, reproducibility, and reduction in the risk of bias. The protocol was initially submitted to PROSPERO; however, due to temporary limitations imposed by the registry, it was subsequently archived and made publicly available via the Open Science Framework (OSF) platform (OSF reference to be added in the final version). The review itself was conducted in accordance with the PRISMA 2020 guidelines [14].

**Search strategy**

The literature search was conducted through a systematic consultation of scientific electronic databases relevant to health sciences and psychology, including PubMed, Scopus, CINAHL, Embase, PsycINFO, and the Cochrane Central Register of Controlled Trials (CENTRAL). Searches were performed from database inception to January 2025. The full search strategies for each database are reported in Appendix A.

**Inclusion and Exclusion Criteria**

Only quantitative studies, randomized and non-randomized, evaluating the application of EW in healthcare professionals or healthcare students were included. Accordingly, both controlled and uncontrolled quantitative studies were considered eligible, provided that they examined a structured EW intervention and reported at least one outcome relevant to psychological health, psychophysical well-being, or work-related functioning. Qualitative and theoretical literature was not included among the studies reviewed, but was considered only for interpretative and contextualization purposes in the Discussion section. For methodological reasons, stringent inclusion and exclusion criteria were applied. Only studies involving a clearly defined and structured EW protocol, consistent with the paradigm originally described by Pennebaker [4], were included. Therefore, interventions based on related but conceptually distinct approaches—such as narrative medicine, reflective writing, journaling, or autobiographical writing—were excluded unless they were explicitly based on a defined EW protocol. Studies not available in full text, publications in languages other than English or Italian, and studies not focused on the population of interest were also excluded.

**Study selection process**

**Figure 1:**
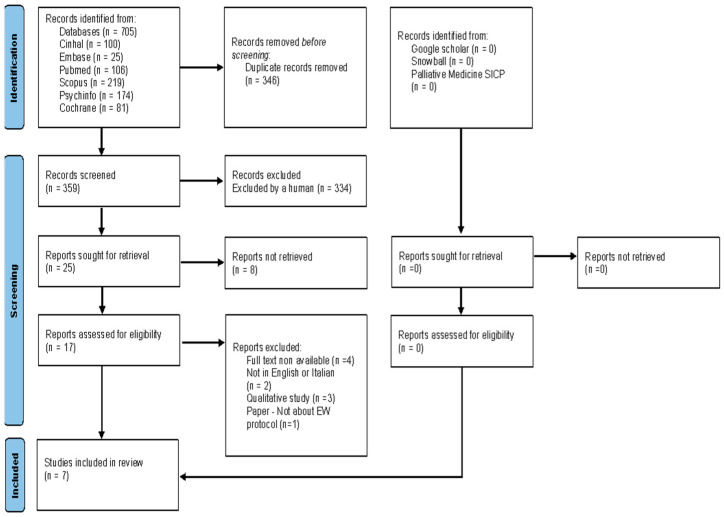
PRISMA flowchart of the study selection process.

The selection process was conducted using Rayyan software [15]. In the first phase, five independent reviewers screened titles and abstracts in a blinded manner to reduce the risk of selection bias. In the second phase, the full texts of potentially eligible articles were assessed for final inclusion. Any disagreements were to be resolved through the involvement of two senior researchers; however, no conflicts occurred during the selection process. The entire selection process was documented using the PRISMA 2020 flow diagram (Figure 1), which led to the final inclusion of seven studies

**Data extraction and outcomes**

Data extraction was conducted systematically and included information on year of publication, geographical area, study design, sample characteristics, intervention characteristics, outcomes assessed, and main findings. The outcomes were grouped into three broad domains: (1) psychological symptoms; (2) indicators of professional and organizational satisfaction; and (3) indicators of psychophysical well-being.

**Data synthesis**

Due to substantial methodological and clinical heterogeneity across the included studies—including differences in study design, participant populations, intervention structure, comparator conditions, and outcome measures—a quantitative meta-analysis was not considered appropriate. Therefore, findings were synthesized narratively. The narrative synthesis was structured to summarize and compare studies according to the (1) study design and sample characteristics; (2) key features of the EW intervention, including number and duration of sessions; and (3) the main outcome domains assessed, with attention to the direction and consistency of findings across studies. This approach was adopted to facilitate cross-study comparison while preserving important methodological differences.

**Assessment of methodological quality and risk of bias**

The methodological quality and risk of bias of the included studies were assessed using different tools according to study design. Specifically, the Cochrane Risk of Bias 2 (RoB 2) tool [16] was used for randomized controlled trials, whereas the Newcastle–Ottawa Scale (NOS) [17] was used for non-randomized studies. Assessments were conducted independently by the reviewers and summarized in dedicated tables.

**Data availability**

Extracted data, summary tables, quality assessments, and risk-of-bias evaluations were systematically organized and made available to support the transparency and replicability of the review.

## 3. Results

As shown in Figure 1, the database search yielded 705 records. After initial screening in Rayyan, duplicate records were removed, reducing the number of records to 359. Following title and abstract screening by the five reviewers, 342 records were excluded. Of the 17 full-text articles assessed for eligibility, 4 were excluded because the full text could not be retrieved, 2 because they were not written in English or Italian, 3 because they were qualitative studies, and 1 because it did not evaluate a structured EW protocol. Additional screening of reference lists and grey literature did not identify any further eligible studies. Seven studies met the inclusion criteria and were included in the review. Study characteristics are summarized in Supplementary Table S1, submitted separately with the manuscript. 

### 3.1. Characteristics of Included Studies

The seven included studies were published between 2017 and 2023. Most of the studies were conducted in Europe, with five studies from Italy [1,7,8,9,10] and two from the United States [11,12]. Across the seven included studies, the nominal sample size reported by the authors totaled 1446 healthcare professionals. However, participant flow varied substantially across studies, and the number of participants who initiated the intervention, completed the writing protocol, or were included in post-intervention analyses was not always consistently reported. Despite this variability, the most represented professional groups included nurses [1,7,8,9,10,11,12], psychologists [7,8,9], psychotherapists [1], physicians, and auxiliary healthcare staff [1,7,9,10]. Cosentino et al. [8] and Tonarelli et al. [7] examined healthcare professionals working in palliative care units, whereas Holliday et al. [12] focused on staff working in a neurosurgical intensive care unit. Regarding sex distribution, women represented the majority of participants in all studies in which this information was reported; in Holliday et al. [12], participant sex was not specified. Mean participant age varied across studies: it was 46 years in Procaccia et al. [10] and Tonarelli et al. [7], 44.74 years in Cosentino et al. [9], and 45.47 years in Tonarelli et al. [1]. In addition, Cosentino et al. [8] categorized participants into four age groups: 18–25 years, 26–35 years (38%; n = 25), 36–45 years, and 46–65 years.

### 3.2. Methodological Quality

**Figure 2:**
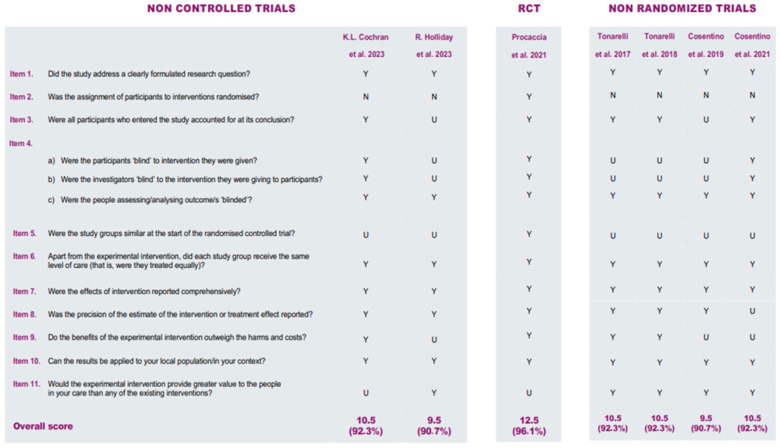
Summary of CASP checklists of included articles [1,7,8,9,10,11,12].

**Figure 3:**
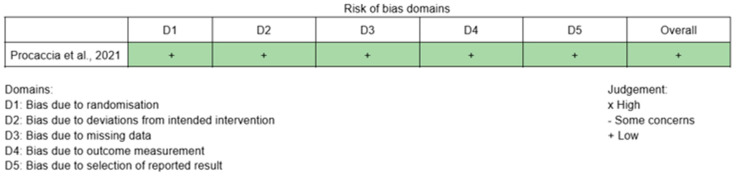
Risk of bias RoB2 scale of RCT article [10].

**Figure 4:**
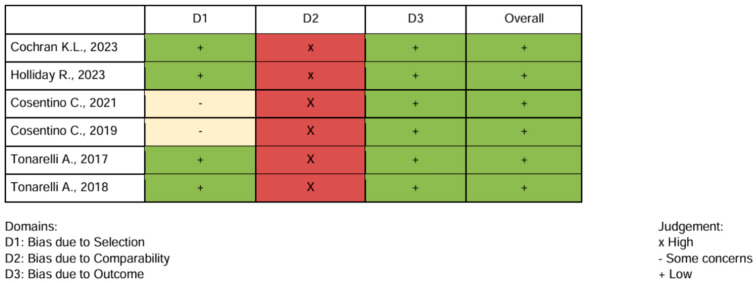
Risk of bias Newcastle–Ottawa scale of non controlled and non randomized trials [1,7,8,9,11,12].

The methodological quality of the included studies was assessed using the Critical Appraisal Skills Programme (CASP). In particular, the Randomised Controlled Trial (RCT) Checklist was applied to assess the overall methodological quality of the included studies, and all studies were found to have good methodological quality (Figure 2). The risk of bias was assessed by the five independent reviewers using the Cochrane Risk of Bias 2 (RoB 2) tool [16] for the randomized trial [10] and the Newcastle–Ottawa Scale [17] for the non-randomized studies [1,7,8,9,11,12] (Figure 3 and Figure 4). 

### 3.3. Interventions

All studies required participants to express their deepest emotions, thoughts and feelings following an event of considerable emotional impact related to their professional experience. Participants had the opportunity to carry out expressive writing sessions at home; in the studies of Holliday et al. [12] and Cochran et al. [11], the intervention was applied using a dedicated online platform and included five sessions of expressive narrative writing (NEW) carried out on a weekly basis. In the studies of Tonarelli et al. [1], Tonarelli et al. [7] and Cosentino et al. [8], an expressive writing protocol was applied, divided into two sessions, with a minimum interval of 1 and a maximum of 3 days. The study by Cosentino et al. [9] involved a first expressive writing session, followed by a further three sessions after a period of 3 weeks. Finally, the study by Procaccia et al. [10] included a single writing session lasting three consecutive days. In all studies, the duration of the writing sessions is between 15 and 30 min.

The results of the included studies were organized into three main outcome domains: (i) psychological symptoms; (ii) indicators of professional and organizational satisfaction; and (iii) indicators of psychophysical well-being.

### 3.4. General Findings

Overall, the quantitative findings suggest that expressive writing may have selective rather than uniform effects across outcome domains in healthcare professionals. The most consistent benefits were observed for psychological symptoms, particularly perceived stress, depressive symptoms, and trauma-related distress, although findings were not uniform across all studies and appeared to be influenced by study design, outcome measures, and attrition. By contrast, professional and organizational outcomes showed more modest and domain-specific improvements, mainly involving selected aspects of job satisfaction, relational functioning, and organizational commitment. Psychophysical and broader well-being outcomes were generally more heterogeneous, with some improvements in coping-related or subjective emotional indicators, but limited or inconsistent evidence for resilience, social support, compassion satisfaction, or broader adaptive functioning. For clarity, results are presented according to three main outcome domains: psychological symptoms; indicators of professional and organizational satisfaction; and indicators of psychophysical well-being.

### 3.5. Psychological Symptoms

Overall, the effects of expressive writing on psychological symptoms appeared more evident for perceived stress, depressive symptoms, and trauma-related outcomes, whereas findings for anxiety and classical burnout dimensions were more heterogeneous. For clarity, psychological symptoms were organized into three subsections: stress; burnout and compassion fatigue; and depressive, anxiety, and post-traumatic stress symptoms.

#### 3.5.1. Stress

This subsection specifically focuses on perceived stress and broader stress-related burden. These outcomes reflect a general and immediate perception of emotional strain rather than occupational burnout or broader psychopathological symptoms. Overall, findings were mixed, with more consistent improvements emerging when direct and standardized stress measures were used. Holliday et al. [12] reported a significant reduction in perceived stress among participants who completed the five-session online NEW program, with mean stress scores decreasing from 50.88 to 31.70 (*p* < 0.05; d = 0.88). In the same study, 58 healthcare professionals initiated the intervention and 31 completed all sessions. By contrast, Cochran and Mealer [11] did not find a statistically significant reduction in perceived stress. In wave 1, mean stress scores decreased from 66.00 to 52.18 (*p* = 0.15), whereas in wave 2 they changed from 62.06 to 64.67 (*p* = 0.87). Interpretation of these findings is limited by substantial attrition, as 1103 nurses enrolled, 430 completed at least one session, and only 154 completed all five sessions. Tonarelli et al. [7] did not assess stress using a specific standardized stress scale, but rather through broader emotional and relational indicators. Therefore, this study may suggest changes in stress-related burden, but it does not provide a clearly isolated quantitative effect on perceived stress.

#### 3.5.2. Burnout and Compassion Fatigue

This subsection focuses on occupational manifestations of chronic work-related distress, including emotional exhaustion, depersonalization, reduced professional accomplishment, and compassion-related burden. These outcomes reflect more specific work-related strain than general perceived stress. Overall, findings were selective rather than generalized, with improvements mainly observed in specific dimensions such as depersonalization, whereas broader burnout-related effects were inconsistent.

Tonarelli et al. [1], using the Maslach Burnout Inventory, reported a significant within-group change in depersonalization (χ^2^ = 30.800, *p* = 0.001), suggesting a selective improvement in one burnout-related dimension. By contrast, Holliday et al. [12] did not observe significant changes in emotional exhaustion (25.69 to 24.46), depersonalization (9.99 to 9.97), or personal accomplishment (34.76 to 33.66) following the online NEW program. Similarly, Cosentino et al. [9] did not report significant pre–post differences in compassion fatigue or compassion satisfaction, and Cosentino et al. [8] found no significant overall changes in the professional quality-of-life variables examined. Taken together, these findings suggest that expressive writing may influence selected burnout-related dimensions, particularly depersonalization, rather than producing a consistent reduction across the broader spectrum of burnout or compassion-related outcomes.

#### 3.5.3. Depressive, Anxiety, and Post-Traumatic Stress Symptoms

This subsection addresses broader and clinically relevant psychopathological outcomes, including affective symptoms and trauma-related distress. These indicators extend beyond immediate work-related strain and capture more structured forms of psychological suffering. Overall, this was the domain in which expressive writing showed the most consistent benefits, particularly for depressive symptoms and post-traumatic stress-related outcomes, whereas anxiety appeared less responsive across studies. Procaccia et al. [10] reported significant reductions in post-traumatic stress symptoms (F = 13.725, *p* = 0.002), depressive symptoms (F = 6.123, *p* = 0.028), and global psychopathology as measured by the SCL-90-R Global Severity Index (F = 5.232, *p* = 0.03) in the expressive writing group compared with controls. Holliday et al. [12] also found a significant reduction in depressive symptoms, with mean depression scores decreasing from 3.93 to 3.03 (*p* < 0.05; d = 0.29), whereas anxiety scores did not change significantly (7.39 to 7.32). Cosentino et al. [9] reported significant improvements in trauma-related symptoms assessed through the IES-R, including intrusive thoughts (z = −2.469, *p* = 0.014), hyperarousal (z = −2.717, *p* = 0.007), and total IES score (z = −2.456, *p* = 0.014). Post-test intrusive thoughts were also significantly lower in the expressive writing group than in controls (U = 202, *p* = 0.038). Overall, these findings suggest that expressive writing may be particularly promising for trauma-related distress and depressive symptomatology, while evidence for anxiety reduction remains limited.

### 3.6. Indicators of Professional and Organizational Satisfaction

This section focuses on work-related functioning and professional adjustment, including job satisfaction, organizational commitment, working climate, and relational aspects of professional experience. These outcomes reflect professional and organizational adaptation rather than symptom reduction per se. Overall, findings were heterogeneous and generally modest, with selective improvements emerging in specific relational and organizational dimensions rather than across broader domains. Tonarelli et al. [7] assessed work satisfaction and relational functioning across pre-intervention, post-intervention, and follow-up. Although no broad and stable significant differences emerged across all domains, the study reported a post-treatment improvement in the working time satisfaction domain (U = 699, *p* = 0.044), which was not maintained at follow-up. Tonarelli et al. [1] reported a significant improvement in satisfaction with colleagues, both within the expressive writing group (χ^2^ = 14.000, *p* = 0.016) and in post-test comparisons with controls (U = 232, *p* = 0.001). Cosentino et al. [8], which examined organizational commitment, compassion satisfaction, and working climate, did not find significant pre–post or between-group differences in the main organizational outcomes, although participants generally evaluated the intervention positively.

By contrast, Cosentino et al. [9] reported a significant increase in continuance commitment (z = −3.357, *p* = 0.001). Taken together, these findings suggest that expressive writing may support selected aspects of professional and organizational functioning, especially relational satisfaction and specific forms of organizational commitment, although effects do not appear robust across all work-related outcomes.

### 3.7. Indicators of Psychophysical Well-Being

This section captures broader adaptive functioning and subjective well-being, including coping strategies, resilience, perceived social support, compassion satisfaction, and non-syndromic indicators of emotional burden. These outcomes reflect more general aspects of adjustment rather than specific psychopathological or occupational symptoms. Overall, findings were more heterogeneous and generally weaker than those observed for depressive and trauma-related symptoms, although some improvements emerged in selected subjective or adaptive indicators. Tonarelli et al. [7] reported significant between-group differences in avoidance coping across assessment points, including baseline (U = 39, *p* = 0.049), post-treatment (U = 380, *p* = 0.035), and follow-up (U = 89, *p* = 0.002), suggesting an unstable pattern over time rather than a clearly sustained benefit. Tonarelli et al. [1] also reported significant improvements in avoidance coping within the expressive writing group (χ^2^ = 19.333, *p* = 0.023) and at post-test versus controls (U = 98, *p* = 0.002). In the same study, transcendent coping also changed significantly within the expressive writing group (χ^2^ = 37.600, *p* < 0.001), although this effect was less central to the broader quantitative pattern. Procaccia et al. [10] included resilience and perceived social support among the assessed outcomes, but no significant overall effects of expressive writing were found for either variable. Similarly, Holliday et al. [12] and Cochran and Mealer [11] did not report significant improvements in resilience. In Cochran and Mealer’s work [11], resilience scores in wave 2 changed minimally from 67.03 to 66.83 (*p* = 0.96), whereas in Holliday et al.’s [12] work, resilience scores changed from 73.03 to 74.03 without statistical significance. Compassion satisfaction was examined in the work of Cosentino et al. [8,9], but no statistically significant changes were observed. This domain also includes broader non-syndromic indicators of emotional burden. In particular, Cosentino et al. [9] reported significant improvements in anger (*p* = 0.027), sleep (*p* = 0.023), and help-seeking (*p* = 0.029) on the Emotion Thermometer, suggesting that expressive writing may influence selected subjective indicators of emotional adjustment even when broader resilience-related constructs remain unchanged.

Supplementary Table S1. Summary of included studies: characteristics, intervention features, and main findings.

## 4. Discussion

Healthcare professionals are frequently exposed to stress, burnout, depression, anxiety, and emotional distress, often compounded by end-of-life care, emotionally demanding clinical encounters, and the cumulative burden of caregiving [18]. The healthcare sector presents specific challenges, including complex decision making, high levels of responsibility, emotionally intense situations related to illness and death, documentation burden, and, in some contexts, the risk of violence or abuse [19]. Uncontrollable work-related factors, such as irregular schedules, opaque organizational systems, individualistic workplace cultures, and poor recognition, may further increase the risk of burnout [20,21]. In this context, identifying simple, accessible, and low-cost supportive tools that can be implemented across different levels of care is particularly important.

EW, widely applied in different populations and settings, may represent a promising and accessible strategy to support the psychological adjustment of healthcare professionals [5]. EW facilitates the processing of emotionally salient experiences and has been associated with improvements in emotional regulation, cognitive reorganization, and meaning-making [7,22,23]. Across the studies included in this review, the clearest quantitative benefits emerged for selected psychological outcomes—particularly perceived stress, depressive symptoms, and trauma-related symptoms—whereas findings for burnout, professional and organizational indicators, and broader psychophysical well-being were more heterogeneous and generally less robust [8,9,10,11,12]. Rather than indicating simple inconsistency, this pattern may reflect differences in the nature of the outcomes assessed. More immediate and state-like outcomes may be more responsive to brief narrative and emotional processing interventions, whereas broader constructs such as resilience, compassion satisfaction, or organizational functioning may require longer interventions, stronger contextual support, or structural changes to show measurable improvement.

This perspective may also help explain the mixed findings related to burnout. Burnout is a multidimensional and context-dependent construct, influenced not only by individual distress but also by workload, institutional climate, team functioning, leadership, and role-related demands. It is therefore plausible that a brief individual intervention such as EW may affect selected components—particularly those closer to emotional distancing or subjective burden, such as depersonalization—without producing uniform changes across all burnout dimensions. This interpretation is consistent with the selective effects observed in some of the included studies, in which specific dimensions improved while broader burnout indices remained unchanged.

The relatively stronger findings for depressive and trauma-related symptoms may also be theoretically coherent with the proposed mechanisms of EW. Writing-based interventions may promote emotional exposure, narrative organization of difficult experiences, and cognitive integration of stressful events. These processes are conceptually closer to intrusive thoughts, hyperarousal, emotional overload, and depressive symptomatology than to more distal occupational or organizational outcomes. Accordingly, the more consistent findings in this review were observed for post-traumatic stress symptoms, depressive symptoms, and perceived stress, whereas anxiety, resilience, and broader professional outcomes appeared less consistently responsive. At the same time, the evidence base remains heterogeneous. The included studies differed substantially in intervention format, number of sessions, duration, delivery mode, timing, clinical context, and outcome selection. As such, this review likely captures not a single, uniform EW intervention, but rather a family of related expressive or narrative writing approaches. This variability may partly explain why findings were more consistent in some domains than in others. In particular, stronger effects reported in smaller or more focused studies may reflect greater protocol intensity, better adherence, or a closer match between intervention and outcome, rather than necessarily stronger intrinsic efficacy. A further issue concerns feasibility and implementation. Online and remotely delivered protocols, such as the Narrative Expressive Writing (NEW) formats used in some studies, may improve accessibility and scalability, especially in overstretched healthcare systems. However, the high attrition rates observed in some of these studies suggest that accessibility does not necessarily translate into sustained engagement. In professionals already exposed to high workload and emotional exhaustion, even low-intensity interventions may remain difficult to complete without protected time, organizational support, or stronger implementation strategies. This suggests that EW may be more feasible and potentially more effective when embedded within broader staff-support frameworks rather than offered as a stand-alone optional activity.

Overall, the findings of this review suggest that EW may best be considered a potentially useful adjunctive intervention rather than a comprehensive solution to occupational distress in healthcare professionals. Its low cost, relative simplicity, and adaptability make it attractive as a supportive tool, particularly in emotionally demanding settings such as palliative care or pandemic-related stress exposure. However, the current evidence supports a cautious interpretation: EW may offer selective benefits for proximal psychological symptoms and some coping- or relationship-related dimensions, while broader occupational and organizational outcomes remain less clearly affected.

This review, conducted according to the Cochrane Handbook for Systematic Reviews of Interventions and reported in accordance with the PRISMA 2020 guidelines, and supported by tools such as CASP and RoB 2, aimed to ensure a satisfactory level of methodological rigor. At the same time, the available evidence reinforces the need for cautious interpretation and for further studies. A further relevant aspect concerns the marked gender imbalance across samples, which were predominantly composed of female participants. Although this likely reflects the high representation of nursing staff in several of the included studies, it limits the generalizability of the findings to the broader population of healthcare professionals.

## 5. Limitations

Despite the promising findings observed in some domains, the reviewed EW studies present several important methodological limitations. First, many studies included relatively small samples, which may reduce statistical power and increase the likelihood of unstable or selective findings. Second, several studies used non-randomized, quasi-experimental, or uncontrolled designs, limiting causal inference and making it difficult to distinguish the specific effects of EW from spontaneous improvement, contextual fluctuations, or regression to the mean. Third, the included studies relied predominantly on self-report measures. While common in psychological intervention research, this may introduce common-method bias, social desirability effects, and over- or underestimation of symptom change. This issue is particularly relevant in the present review, in which many outcomes—including stress, burnout, resilience, and emotional burden—were assessed through self-administered scales without objective or observer-based indicators. Fourth, there was substantial heterogeneity in intervention protocols, including differences in writing prompts, number and duration of sessions, timing, and delivery mode. This reduces comparability across studies and complicates efforts to determine which EW format may be most effective for specific outcomes or settings. Similarly, there was marked heterogeneity in the conceptualization and measurement of outcomes, particularly across stress-related, burnout-related, well-being, and organizational constructs. Another relevant limitation concerns adherence and attrition. In some studies—particularly those using online or multi-session formats—dropout rates were substantial. This may introduce selection bias, as participants who complete the intervention may differ systematically from those who discontinue it, potentially inflating observed effects. In addition, follow-up assessments were often absent, brief, or inconsistent, limiting conclusions about the durability of observed benefits. Finally, the generalizability of the findings is constrained by the composition of the samples. Many studies were conducted in specific contexts, such as palliative care or pandemic-related settings, and often involved predominantly female participants and specific professional categories, especially nursing staff. Although these populations are highly relevant, the findings may not be directly transferable to all healthcare professions, care settings, or organizational cultures.

## 6. Conclusions

This systematic review aimed to investigate the application of EW among healthcare professionals, with particular attention to psychological symptoms, psychophysical well-being, and perceived professional and organizational adjustment. Overall, the available evidence suggests that EW may represent a promising and accessible supportive strategy in healthcare settings, particularly for facilitating emotional processing and the re-elaboration of stressful or emotionally demanding work experiences [4,5]. Across the included studies, the most consistent quantitative benefits were observed for selected psychological outcomes, especially perceived stress, depressive symptoms, and trauma-related symptoms. By contrast, findings for burnout, resilience, compassion satisfaction, and broader professional or organizational outcomes were more variable and less conclusive [8,9,10,11,12]. These findings suggest that EW may be more useful for proximal outcomes closely linked to emotional processing than for broader and more stable dimensions of occupational well-being.

At the same time, the current evidence base remains limited by small sample sizes, frequent use of non-controlled or non-randomized designs, heterogeneity in protocols and outcome measures, and the predominant reliance on self-report instruments. Low adherence and attrition, especially in online and multi-session formats, further highlight the need for implementation models that are both feasible and acceptable within real-world healthcare environments. From a practical perspective, EW should not be considered a stand-alone solution to occupational distress in healthcare professionals. Rather, it may be better understood as a low-cost, flexible, and potentially scalable adjunctive intervention that could be integrated into broader staff-support or well-being programs, particularly in emotionally demanding settings. Future research should prioritize larger and more diverse samples, controlled and randomized designs, longer follow-up periods, and greater standardization of intervention protocols and outcome definitions. It would also be valuable to clarify whether EW is more effective in specific subgroups of healthcare professionals, in particular clinical contexts, or when targeted to participants with higher baseline distress. In addition, future studies should further examine implementation-related variables such as engagement, adherence, protected time, and organizational support. Taken together, the current evidence supports a cautious but clinically relevant interpretation: EW may offer meaningful benefits for selected psychological outcomes in healthcare professionals, but stronger and more methodologically robust evidence is needed before broader conclusions can be drawn regarding its role in occupational well-being or organizational functioning.

## Data Availability

No new data were created or analyzed in this study. Data sharing is not applicable to this article.

## Supplementary Materials

The following supporting information can be downloaded at: https://www.mdpi.com/article/10.3390/healthcare14081057/s1, Table S1: Characteristics of Included articles.

## Appendix A

**Table A1:** Search strategies for each database.

PubMed	(“Health Personnel”[Majr] OR “Allied Health Personnel”[Majr] OR “Dentists”[Majr] OR “Physicians”[Majr] OR “Nurses”[Majr] OR “Nurse Midwives”[Majr] OR “Nutritionists”[Majr] OR “Occupational Therapists”[Mesh] OR “Optometrists”[Majr] OR “Pharmacists”[Majr] OR “Physical Therapists”[Majr] OR “Psychotherapists”[Majr] OR “Healthcare Worker*”[Title/Abstract] OR “Health care Worker*”[Title/Abstract] OR “HealthCare Provider*”[Title/Abstract] OR “Health care Provider*”[Title/Abstract] OR “HealthCare Profession*”[Title/Abstract] OR “health care Profession*”[Title/Abstract] OR “healthcare practitioner*”[Title/Abstract] OR “health care practitioner*”[Title/Abstract] OR “Allied Health Personnel”[Title/Abstract] OR “paramedical profession*”[Title/Abstract] OR “audiologist*”[Title/Abstract] OR “Dentist*”[Title/Abstract] OR “paramedic*”[Title/Abstract] OR “Physician*”[Title/Abstract] OR “doctor*”[Title/Abstract] OR “practitioner*”[Title/Abstract] OR “nurs*”[Title/Abstract] OR “Nutritionist*”[Title/Abstract] OR “Dietician*”[Title/Abstract] OR “Dietitian*”[Title/Abstract] OR “Occupational Therapist*”[Title/Abstract] OR “Optometrist*”[Title/Abstract] OR “optometric technician*”[Title/Abstract] OR “Pharmacist*”[Title/Abstract] OR “Physical Therapist*”[Title/Abstract] OR “Physiotherapist*”[Title/Abstract] OR “Psychotherapist*”[Title/Abstract] OR “psychologist*”[Title/Abstract] OR “Midwi*”[Title/Abstract] OR “radiographer*”[Title/Abstract] OR “speech therap*”[Title/Abstract]) AND (“expressive writing”[Title/Abstract] OR “therapeutic writing”[Title/Abstract] OR “emotional writing”[Title/Abstract] OR “emotional upheavals”[Title/Abstract] OR “emotional disclosure”[Title/Abstract]
Scopus	(TITLE-ABS-KEY ((“Healthcare Worker*” OR “Health care Worker*” OR “HealthCare Provider*” OR “Health care Provider*” OR “HealthCare Profession*” OR “health care Profession*” OR “healthcare practitioner*” OR “health care practitioner*” OR “Allied Health Personnel” OR “paramedical profession*” OR “audiologist*” OR “Dentist*” OR “paramedic*” OR “Physician*” OR “doctor*” OR “practitioner*” OR “nurs*” OR “Nutritionist*” OR “Dietician*” OR “Dietitian*” OR “Occupational Therapist*” OR “Optometrist*” OR “optometric technician*” OR “Pharmacist*” OR “Physical Therapist*” OR “Physiotherapist*” OR “Psychotherapist*” OR “psychologist*” OR “Midwi*” OR “radiographer*” OR “speech therap*”))) AND (TITLE-ABS-KEY ((“expressive writing” OR “therapeutic writing” OR “emotional writing” OR “emotional upheavals” OR “emotional disclosure”)))
Cinahl	MM “Health Personnel” MM “Allied Health Personnel” MM “Dentists” MM “Physicians” MM “Nurses” MM “Midwives” MM “Certified Nurse Midwives” MM “Nutritionists” MM “Dietitians MM “Occupational Therapists” MM “Optometrists” MM “Pharmacists” MM “Physical Therapists” MM “Psychotherapists” MM “Psychologists” MM “Audiologists” MM “Speech-Language Pathologists” TI (“Healthcare Worker*” OR “Health care Worker*” OR “HealthCare Provider*” OR “Health care Provider*” OR “HealthCare Profession*” OR “health care Profession*” OR “healthcare practitioner*” OR “health care practitioner*” OR “Allied Health Personnel” OR “paramedical profession*” OR “audiologist*” OR “Dentist*” OR “paramedic*” OR “Physician*” OR “doctor*” OR “practitioner*” OR “nurs*” OR “Nutritionist*” OR “Dietician*” OR “Dietitian*” OR “Occupational Therapist*” OR “Optometrist*” OR “optometric technician*” OR “Pharmacist*” OR “Physical Therapist*” OR “Physiotherapist*” OR “Psychotherapist*” OR “psychologist*” OR “Midwi*” OR “radiographer*” OR “speech therap*”) AB (“Healthcare Worker*” OR “Health care Worker*” OR “HealthCare Provider*” OR “Health care Provider*” OR “HealthCare Profession*” OR “health care Profession*” OR “healthcare practitioner*” OR “health care practitioner*” OR “Allied Health Personnel” OR “paramedical profession*” OR “audiologist*” OR “Dentist*” OR “paramedic*” OR “Physician*” OR “doctor*” OR “practitioner*” OR “nurs*” OR “Nutritionist*” OR “Dietician*” OR “Dietitian*” OR “Occupational Therapist*” OR “Optometrist*” OR “optometric technician*” OR “Pharmacist*” OR “Physical Therapist*” OR “Physiotherapist*” OR “Psychotherapist*” OR “psychologist*” OR “Midwi*” OR “radiographer*” OR “speech therap*”) #1 OR #2 OR #3 OR #4 OR #5 OR #6 OR #7 OR #8 OR #9 OR #10 OR #11 OR #12 OR #13 OR #14 OR #15 OR #16 OR #17 OR #18 OR #19 TI (“expressive writing” OR “therapeutic writing” OR “emotional writing” OR “emotional upheavals” OR “emotional disclosure”) AB (“expressive writing” OR “therapeutic writing” OR “emotional writing” OR “emotional upheavals” OR “emotional disclosure”) #21 OR #22 #20 AND #23
PsychInfo	MM “Health Personnel” MM “Allied Health Personnel” MM “Dentists” MM “Physicians” MM “Nurses” MM “Nutritionists” MM “Occupational Therapists” MM “Optometrists” MM “Pharmacists” MM “Physical Therapists” MM “Psychotherapists” MM “Psychologists” MM “Speech Therapists” TI (“Healthcare Worker*” OR “Health care Worker*” OR “HealthCare Provider*” OR “Health care Provider*” OR “HealthCare Profession*” OR “health care Profession*” OR “healthcare practitioner*” OR “health care practitioner*” OR “Allied Health Personnel” OR “paramedical profession*” OR “audiologist*” OR “Dentist*” OR “paramedic*” OR “Physician*” OR “doctor*” OR “practitioner*” OR “nurs*” OR “Nutritionist*” OR “Dietician*” OR “Dietitian*” OR “Occupational Therapist*” OR “Optometrist*” OR “optometric technician*” OR “Pharmacist*” OR “Physical Therapist*” OR “Physiotherapist*” OR “Psychotherapist*” OR “psychologist*” OR “Midwi*” OR “radiographer*” OR “speech therap*”) AB (“Healthcare Worker*” OR “Health care Worker*” OR “HealthCare Provider*” OR “Health care Provider*” OR “HealthCare Profession*” OR “health care Profession*” OR “healthcare practitioner*” OR “health care practitioner*” OR “Allied Health Personnel” OR “paramedical profession*” OR “audiologist*” OR “Dentist*” OR “paramedic*” OR “Physician*” OR “doctor*” OR “practitioner*” OR “nurs*” OR “Nutritionist*” OR “Dietician*” OR “Dietitian*” OR “Occupational Therapist*” OR “Optometrist*” OR “optometric technician*” OR “Pharmacist*” OR “Physical Therapist*” OR “Physiotherapist*” OR “Psychotherapist*” OR “psychologist*” OR “Midwi*” OR “radiographer*” OR “speech therap*”) #1 OR #2 OR #3 OR #4 OR #5 OR #6 OR #7 OR #8 OR #9 OR #10 OR #11 OR #12 OR #13 OR #14 OR #15 TI (“expressive writing” OR “therapeutic writing” OR “emotional writing” OR “emotional upheavals” OR “emotional disclosure”) AB (“expressive writing” OR “therapeutic writing” OR “emotional writing” OR “emotional upheavals” OR “emotional disclosure”) #17 OR #18 #16 AND #19
Embase	(‘health care personnel’/mj OR ‘audiologist’/mj OR ‘paramedical personnel’/mj OR ‘dentist’/mj OR ‘physician’/mj OR ‘nurse’/mj OR ‘dietitian’/mj OR ‘occupational therapist’/mj OR ‘optometrist’/mj OR ‘pharmacist’/mj OR ‘physiotherapist’/mj OR ‘psychotherapist’/mj OR ‘psychologist’/mj OR ‘midwife’/mj OR ‘radiographer’/mj OR ‘healthcare worker*’:ti,ab,kw OR ‘health care worker*’:ti,ab,kw OR ‘healthcare provider*’:ti,ab,kw OR ‘health care provider*’:ti,ab,kw OR ‘healthcare profession*’:ti,ab,kw OR ‘health care profession*’:ti,ab,kw OR ‘healthcare practitioner*’:ti,ab,kw OR ‘health care practitioner*’:ti,ab,kw OR ‘allied health personnel’:ti,ab,kw OR ‘paramedical profession*’:ti,ab,kw OR ‘audiologist*’:ti,ab,kw OR ‘dentist*’:ti,ab,kw OR ‘paramedic*’:ti,ab,kw OR ‘physician*’:ti,ab,kw OR ‘doctor*’:ti,ab,kw OR ‘practitioner*’:ti,ab,kw OR ‘nurs*’:ti,ab,kw OR ‘nutritionist*’:ti,ab,kw OR ‘dietician*’:ti,ab,kw OR ‘dietitian*’:ti,ab,kw OR ‘occupational therapist*’:ti,ab,kw OR ‘optometrist*’:ti,ab,kw OR ‘optometric technician*’:ti,ab,kw OR ‘pharmacist*’:ti,ab,kw OR ‘physical therapist*’:ti,ab,kw OR ‘physiotherapist*’:ti,ab,kw OR ‘psychotherapist*’:ti,ab,kw OR ‘psychologist*’:ti,ab,kw OR ‘midwi*’:ti,ab,kw OR ‘radiographer*’:ti,ab,kw OR ‘speech therap*’:ti,ab,kw) AND (‘expressive writing’/mj OR ‘expressive writing’:ti,ab,kw OR ‘therapeutic writing’:ti,ab,kw OR ‘emotional writing’:ti,ab,kw OR ‘emotional upheavals’:ti,ab,kw OR ‘emotional disclosure’:ti,ab,kw)
CENTRAL	MeSH descriptor: [Health Personnel] explode all trees MeSH descriptor: [Allied Health Personnel] explode all trees MeSH descriptor: [Dentists] explode all trees MeSH descriptor: [Physicians] explode all trees MeSH descriptor: [Nurses] explode all trees MeSH descriptor: [Nurse Midwives] explode all trees MeSH descriptor: [Nutritionists] explode all trees MeSH descriptor: [Occupational Therapists] explode all trees MeSH descriptor: [Optometrists] explode all trees MeSH descriptor: [Pharmacists] explode all trees MeSH descriptor: [Physical Therapists] explode all trees MeSH descriptor: [Psychotherapists] explode all trees Healthcare NEXT Worker* OR Health NEXT care NEXT Worker* OR HealthCare NEXT Provider* OR Health NEXT care NEXT Provider* OR HealthCare NEXT Profession* OR health NEXT care NEXT Profession* OR healthcare NEXT practitioner* OR health NEXT care NEXT practitioner* OR Allied NEXT Health NEXT Personnel OR paramedical NEXT profession* OR audiologist* OR Dentist* OR paramedic* OR Physician* OR doctor* OR practitioner* OR nurs* OR Nutritionist* OR Dietician* OR Dietitian* OR Occupational NEXT Therapist* OR Optometrist* OR optometric NEXT technician* OR Pharmacist* OR Physical NEXT Therapist* OR Physiotherapist* OR Psychotherapist* OR psychologist* OR Midwi* OR radiographer* OR speech NEXT therap* #1 OR #2 OR #3 OR #4 OR #5 OR #6 OR #7 OR #8 OR #9 OR #10 OR #11 OR #12 OR #13 expressive NEXT writing OR therapeutic NEXT writing OR emotional NEXT writing OR emotional NEXT upheavals OR emotional NEXT disclosure #14 AND #15
