# Supplementary material for: The Use of Expressive Writing in Healthcare Professionals: A Systematic Review of Quantitative Studies
Source: Healthcare (Basel). 2026 Apr 16;14(8):1057. doi: 10.3390/healthcare14081057 (PMC13116220; doi:10.3390/healthcare14081057)
Supplement: Supplementary file 1 [file healthcare-14-01057-s001.zip › healthcare-4193942-supplementary.pdf]

Table S1. Characteristics of Included articles [1,7–12].

Title	Authors	Pubblication year	Geaographical area	Design	Sample characteristics	Key findings
Expressive writing. A tool to help health workers. Research project on the benefits of expressive writing	Tonarelli A, Cosentino C, Artioli D, Borciani S, Camurri E, Colombo B, D'Errico A, Lelli L, Lodini L, Artioli G.	2017	Italy	Non randomized trial	Nurses, Psychologists, Doctors, Physiotherapists,health care assistants. (*n*=66)	The avoidance coping strategy was reduced, albeit not significantly from the phase preceding the post-intervention phase of expressive writing. On the other hand, there was a rebound effect in the follow up.
Expressive writing. A tool to help health workers of palliative care	Tonarelli A, Cosentino C, Tomasoni C, Nelli L, Damiani I, Goisis S, Sarli L, Artioli G.	2018	Italy	Non randomized trial	Nurses, Psychologists, health care assistants (*n*= 26)	Significant difference in job satisfaction relating to colleagues. From the comparison within the experimental group, there is a significant difference in a dimension that identifies the burnout
Effects of Expressive Writing on organizational variables in Palliative Care health Professionals: an explorative study	Cosentino C, Corte C, Fioresi E, Ojeda FM, Pastore S, Severgnini F, Spacci A, Artioli G.	2019	Italy	Non randomized trial	Nurses, Health Care Workers, Physicians, and Psychologists (*n*=66)	No significant difference between PRE and POST emerged within the EW group. A slight tendency towards improvement was shown for the more strictly organizational variables. Age has a significant effect on affective commitment.
Effectiveness of expressive writing	Cosentino C, D'apice C, Del	2021	Italy	Non randomized trial	Nurses, social and health workers,	No significant differences were identified on

protocol in palliative care healthworker: a quantitative study	Gaudio M, Bertoletti C, Bini M, Liotti MC, Melli E, Tesa F, Sarli L, Artioli G.				auxiliaries, doctors, psychologists<br><br>(*n*=50)	Resilience, CS, and CF.<br>A significant reduction after Ew intervention is evident in the parameters contained in the Emotions Thermometer
Benefits of Expressive Writing on Healthcare Workers' Psychological Adjustment During the COVID-19 Pandemic	Procaccia R, Segre G, Tamanza G, Manzoni GM	2021	Italy	RCT	Nurses, Physicians, Allied healthcare workers<br><br>(*n*=55)	Statistically significant interaction effects were found for ptsd symptoms, depression symptoms and global severity index.<br>No effects for social support and resilience were found.
An evaluation of a narrative expressive writing program for nurses during the COVID-19 pandemic.	Cochran KL, Mealer M.	2023	United States	Non controlled trial	Nurses<br><br>(*n*= 1103)	Moderate, but not statistically significant, therapeutic effect on stress levels.<br>No significant change in resilience.
Brief narrative writing program implemented in a neurosurgical intensive care unit during the COVID-19 pandemic	Holliday R, Ricke DJ, Ricklefs C, and Mealer M.	2023	United States	Non controlled trial	Nurses, nursing assistants, staff members of neurosurgical unit<br><br>(*n*=80)	Significant reductions in depressive symptoms and perceived stress.<br>Symptoms of anxiety, exhaustion, depersonalization, personal accomplishment and resilience did not significantly change after program completion.
